# Readability of pediatric health materials for preventive dental care

**DOI:** 10.1186/1472-6831-6-14

**Published:** 2006-11-16

**Authors:** Rachel L Hendrickson, Colleen E Huebner, Christine A Riedy

**Affiliations:** 1 Department of Dental Public Health Sciences, School of Dentistry, University of Washington, 1959 NE Pacific St., Box 357475 Seattle, WA, USA; 2 Department of Health Services, School of Public Health and Community Medicine, University of Washington, 1959 NE Pacific St., Box 35723, Seattle, WA, USA

## Abstract

**Background:**

This study examined the content and general readability of pediatric oral health education materials for parents of young children.

**Methods:**

Twenty-seven pediatric oral health pamphlets or brochures from commercial, government, industry, and private nonprofit sources were analyzed for general readability ("usability") according to several parameters: readability, (Flesch-Kincaid grade level, Flesch Reading Ease, and SMOG grade level); thoroughness, (inclusion of topics important to young childrens' oral health); textual framework (frequency of complex phrases, use of pictures, diagrams, and bulleted text within materials); and terminology (frequency of difficult words and dental jargon).

**Results:**

Readability of the written texts ranged from 2^nd ^to 9^th ^grade. The average Flesch-Kincaid grade level for government publications was equivalent to a grade 4 reading level (4.73, range, 2.4 – 6.6); F-K grade levels for commercial publications averaged 8.1 (range, 6.9 – 8.9); and industry published materials read at an average Flesch-Kincaid grade level of 7.4 (range, 4.7 – 9.3). SMOG readability analysis, based on a count of polysyllabic words, consistently rated materials 2 to 3 grade levels higher than did the Flesch-Kincaid analysis. Government sources were significantly lower compared to commercial and industry sources for Flesch-Kincaid grade level and SMOG readability analysis. Content analysis found materials from commercial and industry sources more complex than government-sponsored publications, whereas commercial sources were more thorough in coverage of pediatric oral health topics. Different materials frequently contained conflicting information.

**Conclusion:**

Pediatric oral health care materials are readily available, yet their quality and readability vary widely. In general, government publications are more readable than their commercial and industry counterparts. The criteria for usability and results of the analyses presented in this article can be used by consumers of dental educational materials to ensure that their choices are well-suited to their specific patient population.

## Background

In the U.S., dental care is the most prevalent unmet health need of children [1-3]. Despite a recent decline in childhood dental decay, it is on the rise among children ages 2 to 5 years [4]. Oral health disparities in the U.S. continue to exist, especially for children from poor and culturally diverse backgrounds [1,5,6]. Among children ages 2 to 5 years, 75% of dental caries is found in 8% of the population [7]. If left untreated, childhood caries can lead to problems with eating, speaking, and learning [8]. As cited in the Maternal Child Health fact sheet, "Oral Health and Learning: when children's oral health suffers, so does their ability to learn", the effects of dental pain may be misunderstood by teachers as a behavioral problem [9].

Infancy and childhood are the most dynamic period of dental growth; thus, educating parents about children's dental care is of critical importance during these periods. Both the American Association of Pediatrics (AAP) and the American Association of Pediatric Dentists (AAPD) recommend that children visit a dentist for an oral health risk assessment within 6 months of birth and establish a dental "home" by age 1 [10].

Creating good dental habits is the result of a chain of communication from provider to parent to the pediatric patient. Parents and guardians, as well as other caregiving adults, have primary responsibility for daily care and preventive service and are the stewards for creating, maintaining, and passing along a good oral health routine to their children. To do so requires an understanding of dental development and how to maintain good oral health. The dental profession and its commercial affiliates play primary roles in educating parents. In order to help parents understand the value of early dental care and home oral hygiene, educational messages must be easy to understand and relevant. Materials to communicate oral health information in writing should be created to maximize readability and comprehension and in full recognition that many U.S. adults, including parents, have limited literacy skills.

It is estimated that, in the U.S. at least 40 million adults have below average literacy skills (< 5^th ^grade reading level), and may be unable to read or understand basic written information that most people take for granted [11]. It is likely, given the context-specific nature of health and its technical jargon, that an individual's health literacy lags behind his/her general literacy level. In fact, the Institute of Medicine recently reported that ninety million American adults have difficulty understanding health information and following treatment plans [12]. Consequently, millions of Americans, including millions of parents, may not be able to fully comprehend basic pediatric health information.

A recent report by the Agency for Healthcare Research and Quality (AHRQ) (2004) [13] reviewed studies of the impact of low health literacy on health and health care utilization. The authors found low literacy was associated with higher use of expensive care, emergency services, and increased rates of hospitalizations. Similarly, a report of the National Work Group (NWG) [14] on Literacy and Health (1998) concluded that; "(1) poor reading skills are associated with poor health status and high use and costs of health care services, 2) reading skills of at least 1 quarter of adult U.S. population are so limited that written communication with this group may not be effective, 3) when written materials are essential, they should generally be at 5^th ^grade level or lower, and (4) clinicians should verify that patients understand the medical information provided to them."

Despite the current research on medical health literacy, very few studies have examined *oral *health literacy. The scope of the problem of low oral health literacy levels among parents of pediatric patients is introduced by Jackson, who suggests several methods of improving the provider-patient communication, including the use of grade level analyses as a means for pediatric dentists to assess their educational materials [15]. One such study by Alexander [16] analyzed the readability of 24 general dental educational publications. It found that 41.7 percent of the materials were written at grade level higher than 8^th ^grade. A similar study by Kang et al used Alexander's framework for readabililty analysis and investigated the readability of pediatric specific oral health educational materials [21]. We expand this research further by assessing readability as well as thoroughness, textual framework, and terminology of 27 publications from a diverse group of sources: the ADA, government health sources, and commercial organizations. Our analysis of differences in readability by source of the documents (i.e., commercial, industrial, and government) is similar to that of Harwood and Harrison who tested the readability of orthodontic patient informational leaflets [22]. Our approach provides a framework for selecting the most readable and comprehensive pediatric oral health materials based on multiple parameters. The results reported here provide dentists and other oral healthcare providers with an analysis of currently available materials. The framework itself can be used now and in the future to select materials that are both highly readable and thorough in their content.

## Methods

### Pediatric Dental Educational Materials

Twenty-seven pamphlets and brochures were examined in this study. The majority of materials were readily and publicly available at no charge; those with a cost attached could be previewed in PDF format or obtained by mail. They were obtained from various sources including local pediatric dental practices, online websites of commercial sources (i.e. Crest, Oral B, and Colgate), government and industry sources. Many of the publications were available online in PDF format. The majority of the government publications were mailed to the study author (RLH) by request, samples of every publication in this study were available at no charge. Nine publications were offered in additional languages other than English. Of the materials sampled, 22% were from commercial sources; 44% were from government sources, and 33% were from industry sources. The source of each publication is provided in Table 1 below.

**Table 1:** Format and Content Analysis: Physical Attributes

**#**	**Publisher**	**Type**	**Title**	**Physical Attributes**	**Target audience**	**Useful Pictures (Yes/No)**	**Bulleted Text (% of pamphlet)**
1	Colgate	C	Oral health for children	2 pp.; single sheets	parents	No	50%
2	Colgate	C	How do I care for my infant's teeth?	1 pg.; single sheet	parents	No	0%
3	Colgate	C	How do I care for my toddler's teeth?	1 pg.; single sheet	parents	No	0%
4	Crest	C	A parent's guide: caring for children's teeth	10 pp.; booklet**	parents	Yes	30%
5	Crest	C	Children's teeth	2 pp; single sheets	parents	Yes	50%
6	Oral B/Braun	C	Start early, start right	12 pp.; trifold	parents	Yes	17%
7	NV State Health	G	Early childhood caries (cavities) prevention	6 pp.; trifold	parents	Yes	100%
8	CDC	G	Brush up on healthy teeth: simple steps for kids' smiles	1 pg.; single sheet	parents	No	100%
9	CDC	G	Brush up on healthy teeth: a quiz for parents about simple steps for kids' smiles	1 pg.; single sheet	parents	No	100%
10	WA State DSHS	G	Baby bottle tooth decay	6 pp.; trifold**	parents	No	25%
11	WA State DSHS	G	Your baby's teeth	6 pp.; trifold***	parents	No	25%
12	WA State DSHS	G	Your baby's healthy teeth	1 pg.; single sheet***	parents	No	100%
13	WA State DSHS	G	Hey, moms!	1 pg.; single sheet***	parents	No	100%
14	WA State DSHS	G	Hints for a healthy mouth: birth through one year	1 pg., single sheet***	parents	No	25%
15	King Cty Library	G	Teeth: a guide to good oral health	6 pp.; trifold	parents	No	100%
16	NIH/NIDCR	G	Seal out tooth decay: a booklet for parents	10 pp.; booklet	parents	Yes	29%
17	NIH/NIDCR	G	Snack smart for healthy teeth	8 pp.; booklet	parents	No	17%
18	NIH/NIDCR	G	A healthy mouth for your baby	10 pp.; booklet****	parents	No	60%
19	ADA	I	You can prevent early childhood caries	6 pp.; trifold	parents	Yes	0%
20	ADA	I	Tips on teething	4 pp.; trifold	parents	No	0%
21	ADA	I	Good oral health for mother & baby	8 pp.; trifold	mothers	Yes	33%
22	ADA	I	Why baby teeth are important	6 pp.; trifold	parents	No	0%
23	ADA	I	Pregnancy and your oral health	8 pp.; trifold	expectant mothers	Yes	25%
24	ADA	I	Training cups: choose carefully, use temporarily	4 pp.; trifold	parents	Yes	0
25	ADA	I	Thumb sucking, finger sucking, and pacifier use	4 pp.; trifold	parents	Yes	50%
26	ADHA	I	Want some life saving advice? Ask your dental hygienist about proper oral health care for children	2 pp.; single sheets	parents	No	50%
27	WDSF	I	Taking care of your child's baby teeth	6 pp.; trifold****	parents	no	75%

Source Types: C – Commercial, G – Government, I – Industry

** available in English, Spanish, French, Chinese, German and Italian; *** available in English, Spanish, Russian, Vietnamese, Cambodian, Laotian and Mandarin Chinese; **** available in English and Spanish

### Usability Analysis

Our analysis of the "usability" of these educational materials considered 3 sets of attributes: format, content and reading level. The review of format included physical characteristics of the material (i.e., number of pages and shape of the document), the intended audience, use of instructional pictures or drawings, and bulleted text. The review of content assessed the thoroughness of the pamphlet or brochure to provide information on 11 topics germane to the oral health of infants, toddlers and preschool children. The review of readability assessed the reading level of the text and the use of dental jargon.

### Format

The criteria for our analyses of format are presented in Table 1 and defined in turn below.

#### • Physical attributes

The physical attributes of the materials are described in terms of the type of document (e.g., booklet, tri-fold brochure, or single page flyer), and the number of pages.

#### • Intended audience

The readership of each publication was determined. Most pamphlets are written for parents and other caregivers. A few publications were tailored for expectant mothers.

#### • Instructional graphics

The majority of publications contained illustrations, mainly of smiling children and infants, or clip art of teeth, smiles, or toothbrush graphics; however, publications earning a score of "yes" in this category were those with graphics of instructional value. Examples of instructional graphics are illustrations of toothbrushing techniques and visual representation of the amount of toothpaste recommended for brushing children's teeth.

#### • Bulleted text

Effective educational materials written for adults are often formatted in bulleted text. This organizational style adds emphasis and is easier to read [17]. To summarize this aspect of the materials, we report the number of pages with over half the page in bulleted text, divided by the number of total pages of text.

### Thoroughness

Judgments about the thoroughness of the brochures and pamphlets were based on the presence of information about 11 oral health topics. The topics are listed in Table 2 and defined in turn below.

**Table 2:** Format and Content Analysis: Thoroughness

**#**	**Baby gum care**	**Tooth development**	**ECC prevention**	**Infant dental visit**	**Tooth-brushing**	**Toothpaste amount**	**Flossing**	**Fluoride**	**Training cups**	**Sealants**	**Avulsed teeth**
1	--	--	X	--	--	X	X	X	--	X	X
2	X	--	X	--	--	--	--	X	--	--	--
3	--	X	--	X	X	X	--	--	--	--	--
4	X	X	X	X	X	X	X	X	--	X	X
5	X	X	--	X	--	X	--	X	--	X	--
6	--	X	X	--	X	X	X	X	--	X	X
7	X	--	X	X	--	--	--	X	X	--	--
8	X	--	--	--	X	X	--	X	--	--	--
9	X	--	--	--	X	X	--	X	--	--	--
10	X	--	X	X	X	X	--	X	--	--	--
11	X	X	X	X	--	--	--	X	--	--	--
12	--	--	X	--	X	X	--	--	--	--	--
13	--	--	X	--	--	--	--	--	--	--	--
14	X	--	X	--	X	X	--	X	X	--	--
15	X	X	X	X	--	X	--	X	X	X	--
16	--	X	--	--	--	--	--	X	--	X	--
17	--	--	X	--	--	--	--	--	--	--	--
18	X	--	X	X	X	X	--	X	X	--	--
19	X	--	X	X	--	--	--	X	X	--	--
20	--	X	X	X	X	X	--	--	--	--	--
21	X	X	X	X	--	--	--	--	--	--	--
22	X	X	X	X	X	X	X	--	--	--	--
23	--	--	---	--	X	--	--	--	--	--	--
24	--	--	X	X	--	--	--	--	X	--	--
25	--	--	--	--	--	--	--	--	--	--	--
26	X	--	X	X	X	X	X	--	--	X	X
27	X	X	X	X	X	X*	--	X	--	X	--

#### • Baby gum care

Gum care is pertinent during the first six months of life, prior to the eruption of the first tooth. Keeping gums clean can prevent the formation of plaque in erupting teeth. The standard practice is to wipe a baby's gums with a clean gauze pad or clean damp cloth. Publications that described this practice were coded "yes" (tabled as 'X"; see Table 2) for this topic.

#### • Infant dental visit

This category is scored "yes" for the presence of guidelines regarding visiting a dentist within 6 months of the eruption of the first tooth or no later than the baby's 1^st ^birthday.

#### • Toothbrushing

Instruction in "how to" brush young children's teeth earned a "yes" for this category.

#### • Toothpaste amount

Direction for how much toothpaste should be used, regardless of precise wording, was coded "yes" for this category. A common description of quantity is a "pea-sized" amount.

#### • Flossing

A "yes" was scored if the publication mentioned flossing; many publications cited a common rule of thumb for when flossing should begin – when two teeth touch. Ideally, flossing should be practiced after consulting with a dentist.

#### • Fluoride

A score of "yes" in this category required an explanation of what fluoride is (a naturally occurring element which can be easily and effectively added to a water source), and how fluoride is beneficial (it fortifies enamel, making it harder and stronger, therefore conferring healthier primary teeth and aiding in caries prevention).

#### • Training cups

A "yes" in this category indicates the publication informed readers of when and why a child should be weaned from training cups. To protect dental health, weaning is recommended by the child's first birthday, because drinking over long periods of time throughout the day bathes the facial surfaces of the child's anterior teeth in sugary, cariogenic liquids.

#### • Sealants

A description of sealants (e.g., a thin plastic coating for the occlusal surfaces of a child's permanent molars) and how sealants help protect the chewing surface from decay earned a score in this category.

#### • Avulsed teeth

Information about how to handle dental trauma (e.g. when a tooth is knocked out) by placing the tooth in a cup of milk, not cleaning out the socket, and bringing the child and tooth into the dentist for re-implantation was required to earn a score of "yes" in this category.

### Reading Level of Text and Use of Professional Jargon

The reading level of each document was determined using three widely-used measures: Flesch-Kincaid grade level, Flesch Reading Ease, and SMOG reading grade level. Additionally, we reviewed each document for the use of professional jargon. The readability measures are presented in Table 3 and defined below.

**Table 3:** Readability Analyses

**#**	**Jargon (instances)**	**Flesh-Kincaid Grade Level**	**Flesh Reading Ease**	**SMOG Reading Level**
1	4	7.6	68.2	9
2	0	8.2	63.2	9
3	1	8.9	63.0	10
4	11	8	62.2	9
5	6	8.7	55.6	11
6	9	6.9	73.0	9
7	1	3.8	82.5	6
8	0	6.3	76.2	7
9	1	5.6	76.9	8
10	0	4.6	82.1	6
11	0	4.4	83.0	6
12	0	4.7	79.9	6
13	0	2.4	90.5	6
14	0	4.6	84.5	6
15	0	4.6	74.1	9
16	0	5.4	76.4	8
17	0	6.6	72.1	8
18	0	3.7	87.3	6
19	8	4.7	79.9	6
20	5	8.7	64.2	10
21	10	8.7	61.3	10
22	7	9.3	58.3	12
23	16	7.8	67.8	8
24	3	7.3	71.8	8
25	8	6.8	69.9	10
26	16	9.3	60.5	11
27	0	4.0	85.8	5

#### • Flesch-Kincaid (F-K) reading grade level

The Flesch-Kincaid grade level formula is derived from two aspects of written publications: average sentence length (ASL) and average number of syllables per word (ASW). The Flesch-Kincaid reading grade level is calculated by the formula: (.39 × ASL) + (11.8 × ASW) - 15.59. The calculation was made using Microsoft Word 2000; the tool can be found on most word processing programs.

#### • Flesch Reading Ease (FRE)

Flesch Reading Ease uses ASL (average sentence length) and ASW (average syllables per word) to determine reading ease. The formula is: 206.835 - (1.015 × ASL) - (84.6 × ASW); total scores can range from 0 to 100. This calculation was also made using Microsoft Word 2000.

#### • SMOG (Simple Measure of Gobbledygook)

SMOG is a count of polysyllabic words typically used to analyze short documents [19].

#### • Professional jargon

This category refers to the total number of complex terms used within the dental profession. Our list of terms (see table 4) includes those identified by Alexander [16] as well as others present in the materials we studied.

**Table 4:** Professional Jargon in Children's Oral Health Informational Materials

Professional Jargon* (19 instances)
Alignment
Appliance
Antimicrobial
Chronic disease
Dental flossette
Disclosing tablet
Elective
Enamel
Fetus
Gingivitis
Interdental
Laceration
Obstruction
Primary teeth
Reflex
Reimplant
Supplements
Tartar
Tissues

*The "jargon" category displays words that would be unfamiliar to an average adult who had not been exposed to health sciences education. Words that are accompanied by a definition or simple explanation in a material were not counted as jargon

### Data Analyses

Tests for differences by source of publication were examined for the following characteristics: format (% bulleted text), content (number of topics covered of 11 total) and readability (F-K grade level and SMOG). The tests were one-way analysis of variance followed by Tamhane's T2 tests (which do not assume equal variance) to identify significant pairwise differences between means.

## Results

### Format

Twenty-seven publications were reviewed; 21 were available free of charge and 6 were available for a fee. The materials ranged from single page handouts, to tri-fold brochures, to 10- and 12- page booklets. Six used no bulleted text and 6 included bulleted text on all pages. On average, the proportion of pages with bulleted text was .65 (SD = .38) for materials from government sources, .25 (SD = .23) for materials from commercial sources, and .26 (SD = .28) for materials from industry. The test of mean differences in the proportion of pages with bulleted text was statistically significant (F(2,24) = 5.13; p = .01); government publications featured significantly more pages of bulleted text than did commercial sources (p = .04) and more than industry-sponsored publications (p = .04).

### Content Analyses

Content analyses checked for the presence of 11 pediatric-specific topics and recommendations; we did not judge depth of coverage. Less than half of the 27 publications covered more than 50% of the topics of interest, indicating most were not comprehensive. The average number of topics presented in the materials was 4.9 of 11; the mean number of topics was fairly similar for government (M = 4.4; SD = 2.2), commercial (M = 6.2; SD = 2.6) and industry sources (M = 4.6; SD = 2.9). The difference was not statistically significant (p = .36).

Two publications stood out for their thoroughness. One was Crest's booklet: "A parent's guide: caring for your children's teeth", which covered 10 of 11 important topics. Although the text is written at an 8^th ^grade level, it utilizes pictures, bullets, and bold text effectively. A relative weakness of this publication is the high frequency of professional jargon; 11 instances in a 10-page booklet. A strength is the booklet is available in English, Spanish, Chinese, French, German and Italian.

Materials with less breadth but more depth were most often from industry sources. The specificity of information provided by some of these was outstanding. For instance, a parent with a question about weaning children from training cups would benefit greatly from the ADA's pamphlet "Training Cups," which offers advice to "choose carefully, use temporarily." Many of the ADA pamphlets are topic-specific, and provide useful, detailed information. ADA pamphlets were available in sets for a charge, although samples can be downloaded and previewed. Also noteworthy was the ADA's publication "Pregnancy and your Oral Health", one of the few pediatric oral health publications available that provided pre-natal oral health information for women.

### Readability of Text

The reading grade-level equivalents of the 27 publications ranged from 2^nd ^grade to 9^th ^grade. Generally, the lower the Flesch-Kincaid Grade Level measurement, the higher (more readable) the Flesch Reading Ease score, although the correlation is not perfect (i.e., three documents had a Flesch-Kincaid Grade Level of 4.6 but Flesch Reading Ease scores of 82.1, 84.5, and 74.1). The SMOG test consistently rated materials 2 to 3 grade levels higher than did the Flesch-Kincaid analysis.

The reading grade level by Flesch-Kincaid of commercial materials ranged from 7^th ^to 9^th^-grade (6.9 to 8.9); the average was 8^th ^grade (M = 8.1; SD = 0.7). Government-sponsored materials ranged in reading grade level from 2.4 to 6.6; the average was 4.7 (SD = 1.2). Industry sources ranged from 4.0 to 9.3; the average was 7.4 (SD = 1.9). The test for significant differences among mean grade levels was statistically significant (F(2,24) = 14.94; p = .000). Pairwise comparisons showed that, on average, government publications had significantly lower readability demands than either the commercial or industry sources (p = .000 and .01 respectively). Our analyses of the SMOG computations showed a similar pattern (F(2,24) = 7.32; p = .01). The average number of polysyllabic words in materials from government sources was 6.8 (SD = 1.1); whereas the mean for documents from commercial sources was 9.5 (SD = 0.8) and the mean for industry-sponsored materials was 8.9 (SD = 2.3).

The lowest reading-grade level was achieved by an informational flyer entitled "Hey Moms!" created by the Washington State Department of Social and Health Services. Its reading level, based on Flesch-Kincaid, is 2^nd ^grade (2.4). The publication features large, easy-to-read text formatted as bullets. Its primary focus is the prevention of early childhood caries by encouraging positive oral hygiene, e.g., limiting bottle fluids to water, brushing, etc. Another easy-to-read publication was the NIDCR's publication, "A Healthy Mouth for Your Baby." This 10-page booklet registered at a 3^rd ^grade reading level (F-K: 3.7). It used large font; more than half the pages (60%) contained bulleted information, and it included no jargon. "A Healthy Mouth for Your Baby" was also the most thorough source of information for infant oral health care: 7 of 11 topics were covered. The booklet was offered in English and Spanish.

## Discussion

Pediatric oral health materials represent an important link in the chain of communication from dentist to parent to child. Although many educational materials exist, and providers have access to them, the materials vary in terms of content and readability. We found many publications that were adequate in terms of having low literacy demands, offered only limited information. Conversely, many of the more comprehensive publications required higher literacy skills and were, therefore, too difficult for a significant portion of the U.S. population to understand. Educational materials that can not be understood can not be effective. Dental and medical providers should be cognizant of multiple parameters, including readability and coverage of important health topics, when selecting patient educational materials. Moreover, new educational materials should be produced with both sets of parameters in mind.

The present study utilized three measures of readability (the Flesch-Kincaid Grade Level, Flesch Reading Ease, and the SMOG), there are numerous other methods which could have been used and some that were not appropriate for many of our materials. For example, the SAM method (Suitability Assessment of Materials), used by Kang [21] was not appropriate because many of the materials reviewed contained less than 100 words. The Flesch-Kincaid Grade Level, Flesch Reading Ease, and SMOG measures were selected for their simplicity and widespread use. However, these formula-based analyses provide information about only one facet of readability. Formatting of the text e.g., font size, use of bold typeface, use of bullets, simplified sentences, and useful pictures or diagrams all contribute to its general readability. Nursing and pharmacy have been using the Flesch-Kincaid and other formula-based readability analyses for years to help develop more readable patient educational pamphlets; it's time dentistry followed suit [16,18,20].

Our content analyses of the educational materials identified instances of conflicting information as well as specific opportunities for high-quality written information to counteract common media messages. Specifically, most brochures suggested parents use a "pea-sized" amount of toothpaste for cleaning a child's teeth – one pamphlet called for a "rice-sized" amount. In the marketplace, however, amount is represented by a thick, wide ribbon of toothpaste – these mixed messages are confusing. Graphics and photographs can be used effectively to convey the correct information about toothpaste as well as numerous other aspects of oral hygiene (e.g., how to brush teeth). Similar to the studies of Kang [21] and Alexander [16], the current study identified many missed opportunities for oral hygiene instruction. Visual aids to model and reinforce behaviors would improve pediatric-specific dental publications by helping to clarify the written information. Additionally, the effectiveness of paper pamphlets compared to other forms of patient educational materials, e.g., websites, videos, remains to be determined. With new recommendations to bring infants to the dentist within their first six months, and establish a dental "home" by their first year (from the AAPD [25]), research to create effective health educational materials is needed for a new patient group – parents of infants and very young children. Materials that are concise, consistent, and thorough are a simple way to bridge the communication gap between provider, parent, and pediatric patient.

The recognition of health, and oral health literacy, as an important link in patient compliance and overall health is relatively new [12]. In medicine, health literacy guidelines now exist to help improve provider-patient communication; dentistry is not far behind. Research and interest in oral health literacy is burgeoning. And while welcomed, it is yet unknown whether improving patient education materials can improve health outcomes. [23]. Studies have shown that improved population literacy is independently correlated with improved health status [24]. This aspect of health literacy calls for further investigation.

Oral health literacy has important implications for the practice of dentistry. Dentists can choose better suited materials for their patient population based upon the parameters of usability, i.e., readability and content, and can use this and other readability papers to select publications that yield favorable reading grade levels (FKGL <5^th ^grade). One consideration that could aid dentists in selecting suitable publications is the printing of the Flesch-Kincaid grade level on the pamphlet itself, or in the advertisement for the materials. This would be an effective way for authors of oral health educational materials to work together with dental healthcare providers to educate the patient population.

### Limitations

Although the current study investigated several criteria outside the Flesch-Kincaid grade level analyses and SMOG readability measures, classifying a publication's readability remains a challenge, and there are many other tenets of a highly readable document which this study did not examine. Some other parameters to assess might include the use of bright colors, highlighting for emphasis, and advantageous use of white space (so the page appears less dense with text) [18]. A second limitation of this study was the subjective selection of dental jargon and difficult words, within the textual framework. It should be noted, however, that many were words that Alexander [16] had deemed difficult in his readability study.

A third consideration is the selection process for pamphlets. The materials for this study were chosen on the basis of their availability, simulating how attainable publications might be to a dentist or oral healthcare provider interested in finding materials for their patients. The price issue is one drawback of providing literature to patients, and because the authors of the current study received samples of materials free of charge, either because the pamphlet was free or a sample could be downloaded, although availability, not cost, was the main selection criteria in the process of reviewing publications. Viewing of those publications with fees attached is easily accomplished by visiting a website. In this regard, materials can be pre-screened before a provider opts to purchase them.

## Conclusion

The readability and quality of content and formatting varies widely across the numerous readily available pediatric oral health care materials. For the most part, government publications are more readable than their commercial and industry counterparts. The criteria for readability (usability) and results of the analyses presented in this article can be used by consumers of dental educational materials to ensure that their choices are well-suited to their specific pediatric patient population.

## Competing interests

The author(s) declare that they have no competing interests.

## Authors' contributions

RLH carried out the study, participated in the analysis of the materials, and drafted the manuscript. CEH participated in the design and analysis of the study, drafted the manuscript, and conducted the statistical analysis. CAR conceived of the study, and participated in the design and analysis of the materials, and drafted the manuscript. All authors read and approved the final manuscript.

## Pre-publication history

The pre-publication history for this paper can be accessed here:


